# Supplementary material for: The Effects of Ferric Sulfate (Fe2(SO4)3) on the Removal of Cyanobacteria and Cyanotoxins: A Mesocosm Experiment
Source: Toxins (Basel). 2021 Oct 23;13(11):753. doi: 10.3390/toxins13110753 (PMC8619581; doi:10.3390/toxins13110753)
Supplement: Supplementary file 1 [file toxins-13-00753-s001.zip › toxins-1410534-supplementary.pdf]

toxins

MDPI

Supplementary Materials: The Effects of Ferric Sulfate ($Fe_2(SO4)_3$) on the Removal of Cyanobacteria and Cyanotoxins: A Mesocosm Experiment

Kim Thien Nguyen Le, Eyerusalem Goitom, Hana Trigui, Sébastien Sauvé, Michèle Prévost and Sarah Dorner

Table S1. List of cyanobacteria species. Associated toxin information adapted from [1].

No	MB		PLSF		Associated toxin group belonging to genera
	Genera	Species	Genera	Species	
1	*Aphanizomenon*	*A. flos-aquae*<br>*A. issatschenkoi*<br>*A. gracile*	*Aphanizomenon*	*A. flos-aquae*<br>*A. issatschenkoi*	Anatoxin-a (ANA-a), Cylindrospermopsin (CYN), Saxitoxin (STX)
2	*Aphanocapsa*	*A. delicatissima*<br>*A. planctonica*<br>*A. holsatica*	*Aphanocapsa*	*A. delicatissima*<br>*A. planctonica*	
3	*Aphanothece*	*A. clathrata brevis*<br>*A. nidulans*<br>*A. smithii*	*Aphanothece*	*A. clathrata brevis*	
4	*Chroococcus*	*C. limneticus*<br>*C. minimus*<br>*C. prescottii*	*Chroococcus*	*C. limneticus*<br>*C. dispersus*<br>*C. prescottii*	
5	*Coelosphaerium*	*C. kuetzinggianum*	*Coelosphaerium*	*C. kuetzinggianum*	
6	*Dolichospermum*	*D. spiroides*<br>*D. circinalis*<br>*D. planctonicum*	*Dolichospermum*	*D. spiroides*<br>*D. circinalis*<br>*D. planctonicum*<br>*D. mendotae*	Microcystis (MC), Anatoxin-a (ANA-a), Anatoxin-a (S), Saxitoxins (STX)
7	*Merismopedia*	*M. tenuissima*<br>*M. punctata*<br>*M. minima*	*Merismopedia*	*M. tenuissima*<br>*M. punctata*<br>*M. minima*	
8	*Microcystis*	*M. aeruginosa*<br>*M. wesenbergii*	*Microcystis*	*M. aeruginosa*<br>*M. wesenbergii*	Microcystin (MC)
9	*Pseudanabaena*	*P. limnetica*<br>*P. mucicola*	*Pseudanabaena*	*P. limnetica*	

Table S2. Removal effectiveness (%) of taxonomic cell counts of individual cyanobacterial genus after 48 hours (Mean±Standard deviation).

Event	Treat	Total cell counts	*Aphanizomenon*	*Aphanocapsa*	*Aphanothece*	*Chroococcus*	*Coelospharium*	*Dolichospermum*	*Merismopedia*	*Microcystis*	*Pseudanabaena*
**MB September 10-12, 2018**	20 mgFe/L	99.96±0.04	97.84±3.05	100.0±0.00	98.30±0.21	100.0±0.00	NA	99.88±0.04	100.0±0.00	99.97±0.01	99.97±2.81
	35 mgFe/L	99.94±0.04	97.69±1.12	100.0±0.00	97.27±0.11	100.0±0.00	NA	99.81±0.05	100.0±0.00	99.65±0.05	97.75±0.79
**MB September 24-26, 2018**	20 mgFe/L	71.91±5.38	68.55±1.94	89.27±6.55	64.38±2.83	NA	NA	75.22±7.48	100.0±0.00	86.92±2.14	72.69±3.93
	35 mgFe/L	96.39±1.29	80.58±7.53	76.56±2.31	69.09±1.63	NA	NA	98.51±1.19	100.0±0.00	93.98±2.61	71.59±8.54
**MB August 13-15, 2019**	20 mgFe/L	94.27±1.67	93.91±1.64	NA	87.73±6.22	NA	100.0±0.00	97.17±3.14	NA	86.55±1.62	NA
	35 mgFe/L	99.35±0.11	99.26±0.15	NA	96.51±1.44	NA	100.0±0.00	99.97±0.01	NA	100.0±0.00	NA
**PLSF June 26-28, 2019**	20 mgFe/L	85.22±6.43	77.17±8.16	NA	-37.5±1.67	NA	35.11±5.01	84.66±4.07	NA	92.66±8.64	NA
	35 mgFe/L	98.99±1.06	98.66±1.44	NA	88.50±4.58	NA	100.0±0.00	99.03±0.95	NA	95.82±4.36	NA
**PLSF July 24-26, 2019**	20 mgFe/L	51.98±6.21	76.76±4.51	NA	-13.5±1.24	100.0±0.00	19.36±1.14	66.57±4.71	77.5±0.28	66.99±0.95	NA
	35 mgFe/L	99.11±0.11	97.93±0.28	NA	92.65±0.96	100.0±0.00	100.0±0.00	100.0±0.00	100.0±0.00	100.0±0.00	NA
**PLSF August 05-07, 2019**	20 mgFe/L	78.21±6.72	72.22±6.32	NA	79.47±4.98	100.0±0.00	75.86±4.33	77.97±5.04	92.14±3.59	96.51±5.39	NA
	35 mgFe/L	99.72±0.65	98.22±0.65	NA	93.46±2.34	NA	100.0±0.00	100.0±0.00	100.0±0.00	99.97±0.01	NA

Removal effectiveness percentage (%) = $\frac{T0 - T48}{T0} \times 100$.

NA: no value

Table S3. Pairwise Kruskal-Wallis test, showing differences in removal of total cell counts and individual cyanobacterial genus between control mesocosms and mesocosms with dose of 20 mgFe/L, control mesocosms and mesocosms with dose of 35 mgFe/L, mesocosms with dose of 20 mgFe/L and 35 mgFe/L in Missisquoi Bay and Petit-Lac-St-François (*p*-value< 0.05 is significant).

**Species**	***p*-value**			***df***	***chi-squared***	***p*-value**
	control-20 mgFe/L	control-35 mgFe/L	20 mgFe/L-35 mgFe/L			
Dolichospermum	0.002	<0.001	0.001	2	24.12	5.7E-06
Aphanothece	0.018	<0.001	0.003	2	17.81	0.0001
Chroococcus	0.047	0.019	0.018	2	7.49	0.0236
Aphanocapsa	0.084	0.281	0.758	2	5.16	0.0754
Microcystis	<0.001	<0.001	0.013	2	23.68	7.1E-06
Merismopedia	0.009	0.019	0.021	2	8.65	0.0132
Aphanizomenon	0.009	<0.001	0.019	2	13.66	0.0010
Coelosphaerium	0.558	0.043	0.011	2	6.43	0.0401
**Total cell counts**	0.002	<0.001	0.043	2	18.59	9.1E-05

Table S4. Removal effectiveness (%) of total intracellular microcystins (intracellular ΣMCs) and individual intracellular cyanotoxins in mesocosms with dose of 20 mgFe/L and 35 mgFe/L after 48 hours (Mean±Standard deviation). Dominant and representative cyanotoxins are shown.

Event	Treat	ΣMCs	MC-LR	MC-RR	MC-LY	MC-LA	CYN	ANA	APA	APB
MB September 10-12, 2018	20 mgFe/L	99.91±0.15	99.67±0.05	99.70±0.10	60.58±0.51	97.86±0.14	-87.91±0.46	-140±4.34	98.72±2.78	99.09±1.81
	35 mgFe/L	99.92±0.02	99.70±0.09	99.74±0.05	61.57±0.19	98.21±0.41	NA	-150±5.21	98.61±4.56	99.21±1.89
MB September 24-26, 2018	20 mgFe/L	99.65±0.01	99.95±0.02	99.86±1.89	89.43±2.03	99.76±2.92	NA	-23±0.98	80.11±1.23	90.91±1.12
	35 mgFe/L	99.89±0.02	99.89±0.03	99.86±2.11	85.53±1.32	99.71±2.87	NA	-98±2.11	88.31±0.78	90.58±2.78
MB August 13-15, 2019	20 mgFe/L	98.92±1.01	99.15±0.58	99.47±0.04	92.34±1.34	98.06±0.37	43.37±2.35	NA	99.77±1.34	93.21±0.03
	35 mgFe/L	99.70±0.19	99.52±0.01	99.91±0.04	95.39±0.98	96.06±0.24	70.01±1.56	NA	99.29±1.45	99.81±2.01
PLSF June 26-28, 2019	20 mgFe/L	97.97±0.26	98.33±1.50	90.83±0.13	97.81±2.31	97.58±0.05	NA	NA	NA	91.27±1.09
	35 mgFe/L	99.83±1.12	99.96±1.01	89.77±0.05	99.88±1.14	96.42±0.01	NA	NA	42.25±3.45	91.16±0.13
PLSF July 24-26, 2019	20 mgFe/L	99.75±0.07	99.37±0.24	43.92±2.01	98.62±1.07	-44.01±0.03	NA	NA	NA	22.94±2.34
	35 mgFe/L	99.75±0.03	99.90±0.25	58.86±0.19	99.45±0.46	3.65±0.01	NA	NA	NA	33.81±2.23
PLSF August 05-07, 2019	20 mgFe/L	98.96±0.41	99.56±0.32	76.46±0.81	98.57±1.45	-109±0.04	NA	NA	NA	50.86±0.09
	35 mgFe/L	98.05±3.81	99.22±0.69	85.59±0.91	97.26±2.06	-46.98±0.98	-130	NA	NA	72.53±0.81

Removal effectiveness percentage (%) = $\frac{T0 - T48}{T0} \times 100$.

NA: no value

Table S5. Pairwise Kruskal-Wallis test, showing differences in removal of total intracellular microcystins (intracellular ΣMCs) and individual intracellular cyanotoxins between control mesocosms and mesocosms with dose of 20 mgFe/L, control mesocosms and mesocosms with dose of 35 mgFe/L, mesocosms with dose of 20 mgFe/L and 35 mgFe/L in Missisquoi Bay and Petit-Lac-St-François (*p*-value<0.05 is significant).

Toxins	*p*-value			*df*	*chi-squared*	*p*-value
	control-20 mgFe/L	control-35 mgFe/L	20 mgFe/L-35 mgFe/L			
**MC-LR**	<0.001	<0.001	0.386	2	20.12	4.2E-05
**MC-LA**	0.057	0.050	0.603	2	4.76	0.0922
**MC-LY**	<0.001	0.001	0.488	2	14.61	0.0006
**MC-RR**	0.003	0.001	0.453	2	11.31	0.0034
**MC-LF**	0.078	0.021	0.475	2	5.96	0.0501
**MC-LW**	0.356	0.119	0.681	2	2.44	0.2941
**MC-HilR**	0.005	0.004	0.862	2	11.05	0.0039
**MC-YR**	0.073	0.098	0.802	2	4.19	0.1235
**MC-WR**	0.785	0.811	0.335	2	0.56	0.7548
**MC-HtyR**	0.864	0.547	0.381	2	1.15	0.561
**[Asp^3]MC-LR**	0.001	0.001	0.876	2	20.05	4.4E-5
**[Asp^3]MC-RR**	0.423	0.388	0.524	2	0.95	0.619
**CYN**	0.328	0.626	0.684	2	0.91	0.6348
**ANA-a**	0.169	0.054	0.409	2	3.99	0.1354
**AP-A**	0.324	0.129	0.862	2	2.16	0.3385
**AP-B**	0.083	0.024	0.184	2	6.69	0.0334
**ΣMCs**	<0.001	<0.001	0.057	2	24.98	3.7E-06

Table S6. Pairwise Kruskal-Wallis test, showing differences in removal of total extracellular microcystins (extracellular ΣMCs) and individual extracellular cyanotoxins between control mesocosms and mesocosms with dose of 20 mgFe/L, control mesocosms and mesocosms with dose of 35 mgFe/L, mesocosms with dose of 20 mgFe/L and 35 mgFe/L in Missisquoi Bay and Petit-Lac-St-François (*p*-value <0.05 is significant).

**Toxins**	***p*-value**			***df***	***chi-squared***	***p*-value**
	control-20 mgFe/L	control-35 mgFe/L	20 mgFe/L-35 mgFe/L			
**MC-LR**	0.053	0.564	0.113	2	5.10	0.078
**MC-LA**	0.684	0.172	0.148	2	1.36	0.505
**MC-LY**	0.965	0.073	0.231	2	6.28	0.043
**MC-RR**	0.355	0.369	0.488	2	2.51	0.285
**MC-LW**	0.149	0.616	0.514	2	2.11	0.347
**[Asp^3]MC-LR**	0.149	0.569	0.076	2	2.95	0.228
**[Asp^3]MC-RR**	0.088	0.089	0.771	2	4.37	0.112
**CYN**	0.306	0.151	0.094	2	2.15	0.340
**AP-A**	0.056	0.099	0.894	2	2.56	0.227
**AP-B**	0.208	0.089	0.414	2	2.55	0.278
**ΣMCs**	0.225	0.525	0.106	2	7.04	0.029

Table S7. Environmental conditions of lake water samples in control mesocosms at T48 (Mean ± standard deviation).

**Parameters**	**Missisquoi Bay**			**Petit-Lac-St-François**		
	**Event A September 12 2018**	**Event B September 26 2018**	**Event C August 15 2019**	**Event a June 28 2019**	**Event b July 26 2019**	**Event c August 07 2019**
**Chlorophyll-*a* (RFU)**	-	-	64.72 ± 0.59	3.37 ± 0.32	5.63 ± 0.16	7.88 ± 0.17
**Phycocyanin (RFU)**	-	-	169.21 ± 0.34	16.42 ± 0.15	0.87 ± 0.03	6.78 ± 0.16
**pH**	7.47 ± 0.07	-	6.33 ± 0.09	9.93 ± 0.06	8.36 ± 0.07	7.37 ± 0.15
**TDS (mg/L)**	105 ± 0.00	-	140.0 ± 4.24	151.0 ± 5.65	118.5 ± 0.71	121.0 ± 0.00
**Temp (°C)**	22.7 ± 0.17	-	22.09 ± 0.17	27.81 ± 0.41	25.37 ± 0.34	25.25 ± 0.02
**TOC (mg C/L)**	19.97 ± 0.24	5.46 ± 0.00	700.0 ± 23.19	11.39 ± 0.23	10.57 ± 0.21	10.19 ± 0.44
**DOC (mg C/L)**	12.17 ± 0.39	5.08 ± 0.05	73.48 ± 1.45	9.83 ± 0.06	10.07 ± 0.83	9.81 ± 0.07
**TN (mg N/L)**	5.46 ± 0.69	1.68 ± 0.01	6.84 ± 1.78	11.21 ± 1.07	1.01 ± 0.15	1.28 ± 0.05
**TP (µg P/L)**	320.92 ± 4.48	177.49 ± 21.03	2074.60 ± 20.22	603.01 ± 3.17	72.08 ± 5.32	89.29 ± 4.81
**DN (mg N/L)**	2.02 ± 0.001	0.48 ± 0.009	1.56 ± 0.11	0.91 ± 0.25	0.58 ± 0.00	0.63 ± 0.03
**DP (µg P/L)**	40.47 ± 1.17	15.43 ± 0.25	215.01 ± 11.61	108.06 ± 2.22	16.46 ± 1.16	21.81 ± 2.13

Table S8. Environmental conditions of lake water samples in mesocosms with dose of 20 mgFe/L at T48 (Mean ± standard deviation).

Parameters	Missisquoi Bay			Petit-Lac-St-François		
	Event A September 12 2018	Event B September 26 2018	Event C August 15 2019	Event a June 28 2019	Event b July 26 2019	Event c August 07 2019
Chlorophyll-*a* (RFU)	–	-	1.04 ± 0.002	0.08 ± 0.00	1.18 ± 0.01	0.82 ± 0.07
Phycocyanin (RFU)	–	-	33.69 ± 5.21	0.49 ± 0.05	0.54 ± 0.00	1.03 ± 0.05
pH	4.9 ± 0.04	-	5.08 ± 1.01	6.09 ± 0.01	7.70 ± 0.19	5.66 ± 0.02
TDS (mg/L)	138 ± 0.00	-	152.00 ± 0.00	130.00 ± 0.00	139.00 ± 0.00	146.00 ± 0.00
Temp (°C)	21.8 ± 0.01	-	22.95 ± 0.04	23.92 ± 0.02	25.21 ± 0.01	25.03 ± 0.01
TOC (mg C/L)	4.52 ± 0.15	2.22 ± 0.09	23.76 ± 4.52	17.56 ± 2.71	3.81 ± 0.51	3.69 ± 0.11
DOC (mg C/L)	3.21 ± 0.03	2.39 ± 0.03	22.62 ± 1.92	15.10 ± 2.87	3.63 ± 0.24	3.44 ± 0.01
TN (mg N/L)	0.67 ± 0.01	1.29 ± 0.01	4.16 ± 1.01	3.71 ± 0.48	0.55 ± 0.007	0.35 ± 0.009
TP (µg P/L)	8.47 ± 0.35	126.02 ± 14.54	167.73 ± 6.34	130.02 ± 14.49	20.12 ± 1.15	14.73 ± 0.71
DN (mg N/L)	0.64 ± 0.001	0.39 ± 0.01	3.28 ± 0.29	1.28 ± 0.04	0.36 ± 0.02	0.23 ± 0.001
DP (µg P/L)	5.51 ± 0.02	7.14 ± 0.14	60.08 ± 12.79	19.34 ± 1.44	6.72 ± 1.56	3.56 ± 0.21

Table S9. Environmental conditions of lake water samples in mesocosms with dose of 35 mgFe/L at T48 (Mean ± standard deviation).

Parameters	Missisquoi Bay			Petit-Lac-St-François		
	Event A September 12 2018	Event B September 26 2018	Event C August 15 2019	Event a June 28 2019	Event b July 26 2019	Event c August 07 2019
Chlorophyll-*a* (RFU)	–	-	1.05 ± 0.002	0.01 ± 0.00	0.09 ± 0.007	0.01 ± 0.00
Phycocyanin (RFU)	–	-	29.69 ± 1.13	0.05 ± 0.00	0.11 ± 0.00	0.015 ± 0.007
pH	3.95 ± 0.08	-	4.01 ± 0.13	4.47 ± 0.15	4.1 ± 0.02	4.01 ± 0.05
TDS (mg/L)	198 ± 0.00	-	221.5 ± 9.81	285.01 ± 7.12	228.00 ± 0.00	296.50 ± 2.12
Temp (°C)	21.05 ± 0.01	-	22.83 ± 0.12	27.73 ± 0.33	25.26 ± 0.03	24.69 ± 0.33
TOC (mg C/L)	4.01 ± 0.06	2.18 ± 0.09	22.02 ± 0.41	5.68 ± 0.34	2.27 ± 0.31	1.68 ± 0.01
DOC (mg C/L)	3.71 ± 0.01	2.41 ± 0.03	21.07 ± 0.91	7.31 ± 0.10	1.87 ± 0.21	1.87 ± 0.10
TN (mg N/L)	0.67 ± 0.01	1.07 ± 0.02	4.06 ± 0.15	0.95 ± 0.01	0.28 ± 0.007	0.29 ± 0.007
TP (µg P/L)	8.83 ± 0.56	109.97 ± 23.21	146.44 ± 7.81	29.29 ± 0.73	6.58 ± 1.71	4.86 ± 0.82
DN (mg N/L)	0.63 ± 0.001	0.41 ± 0.007	2.93 ± 0.08	0.72 ± 0.04	0.25 ± 0.001	0.27 ± 0.00
DP (µg P/L)	7.11 ± 0.04	6.92 ± 0.04	64.71 ± 4.23	17.99 ± 2.02	4.49 ± 0.11	4.01 ± 0.19

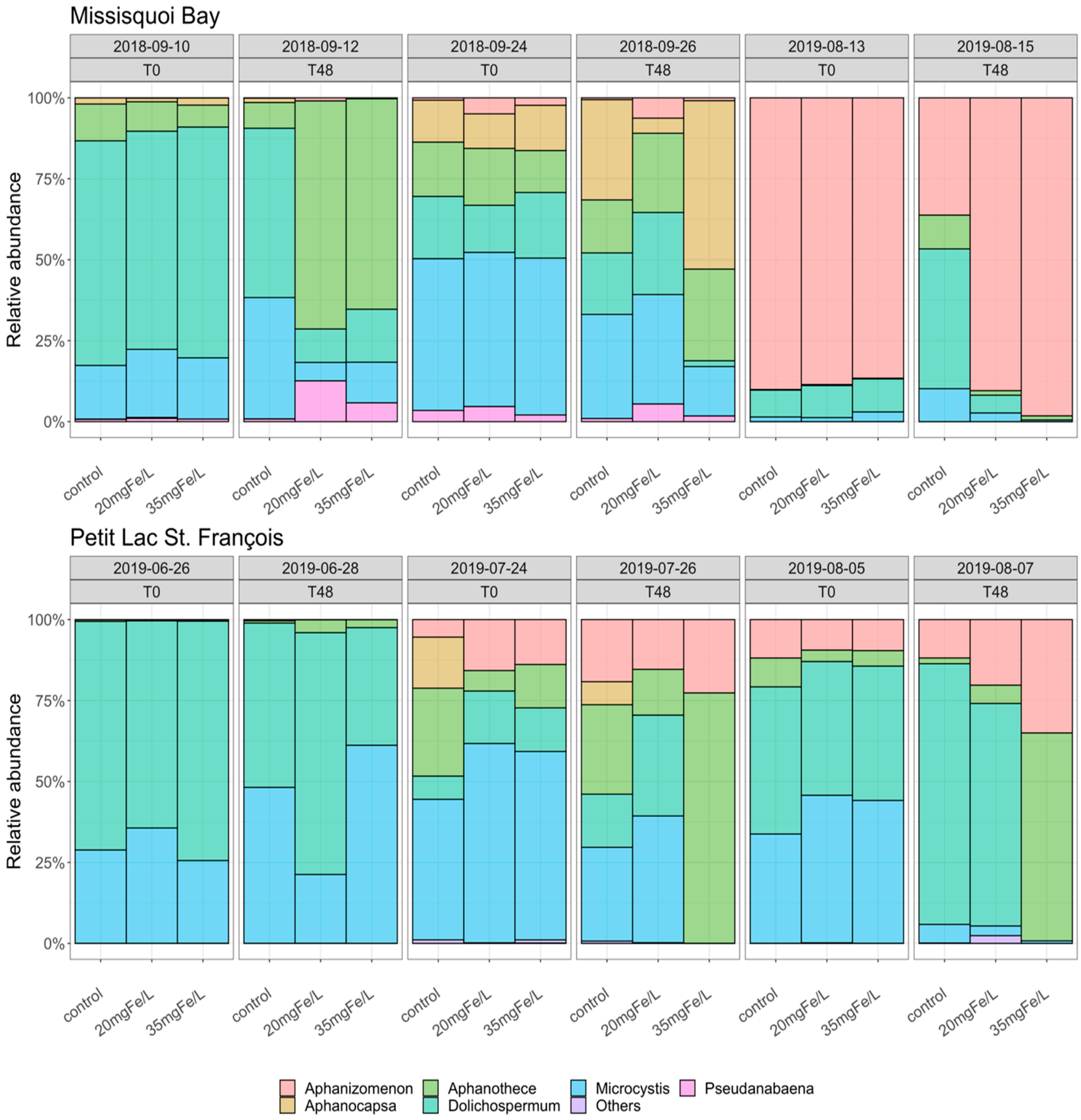

Figure S1. Relative abundance of cyanobacterial cells at genus level.

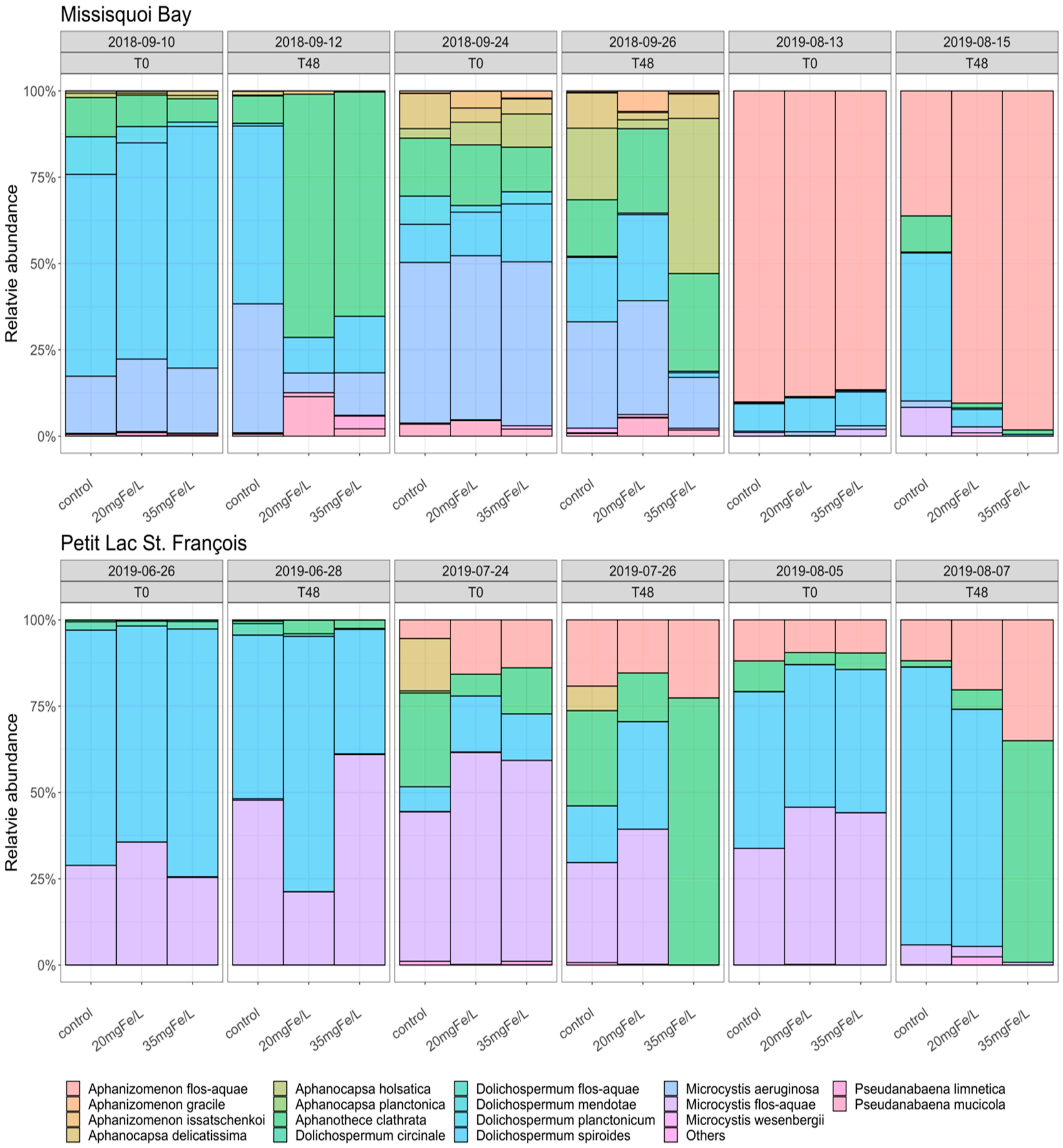

Figure S2. Relative abundance of cyanobacterial cells at species level.

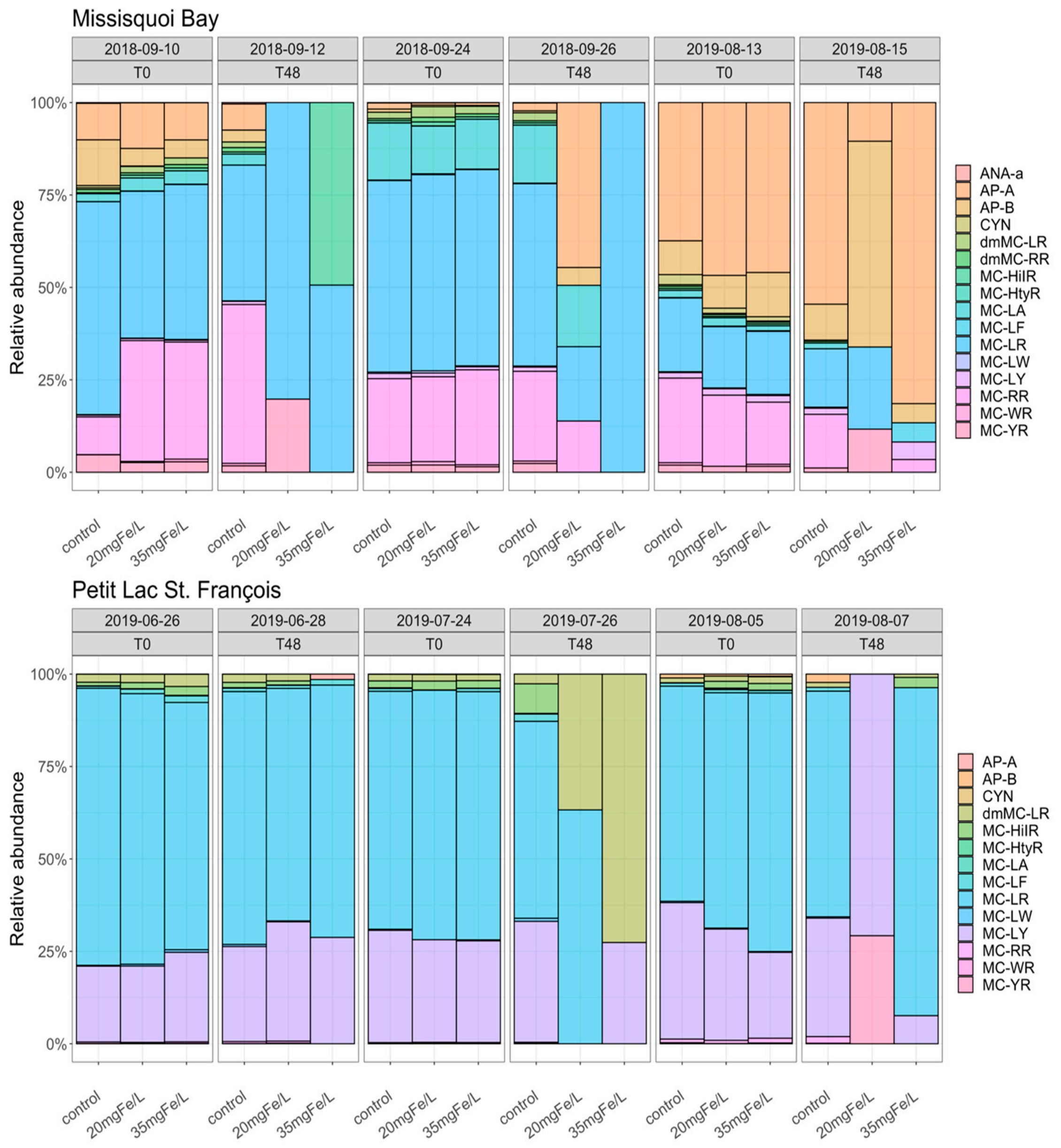

Figure S3. Relative abundance of intracellular cyanotoxins.

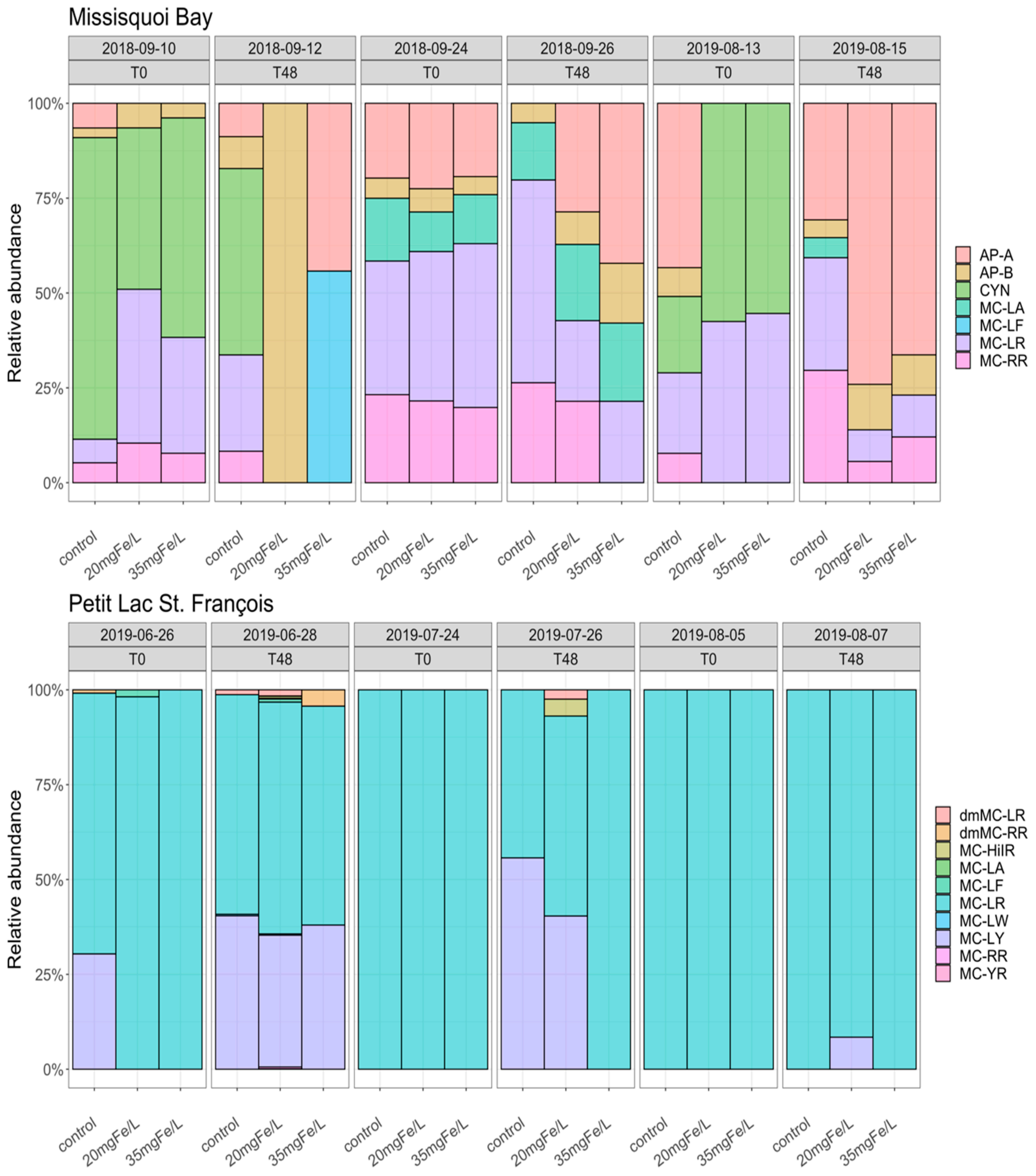

Figure S4. Relative abundance of extracellular cyanotoxins.

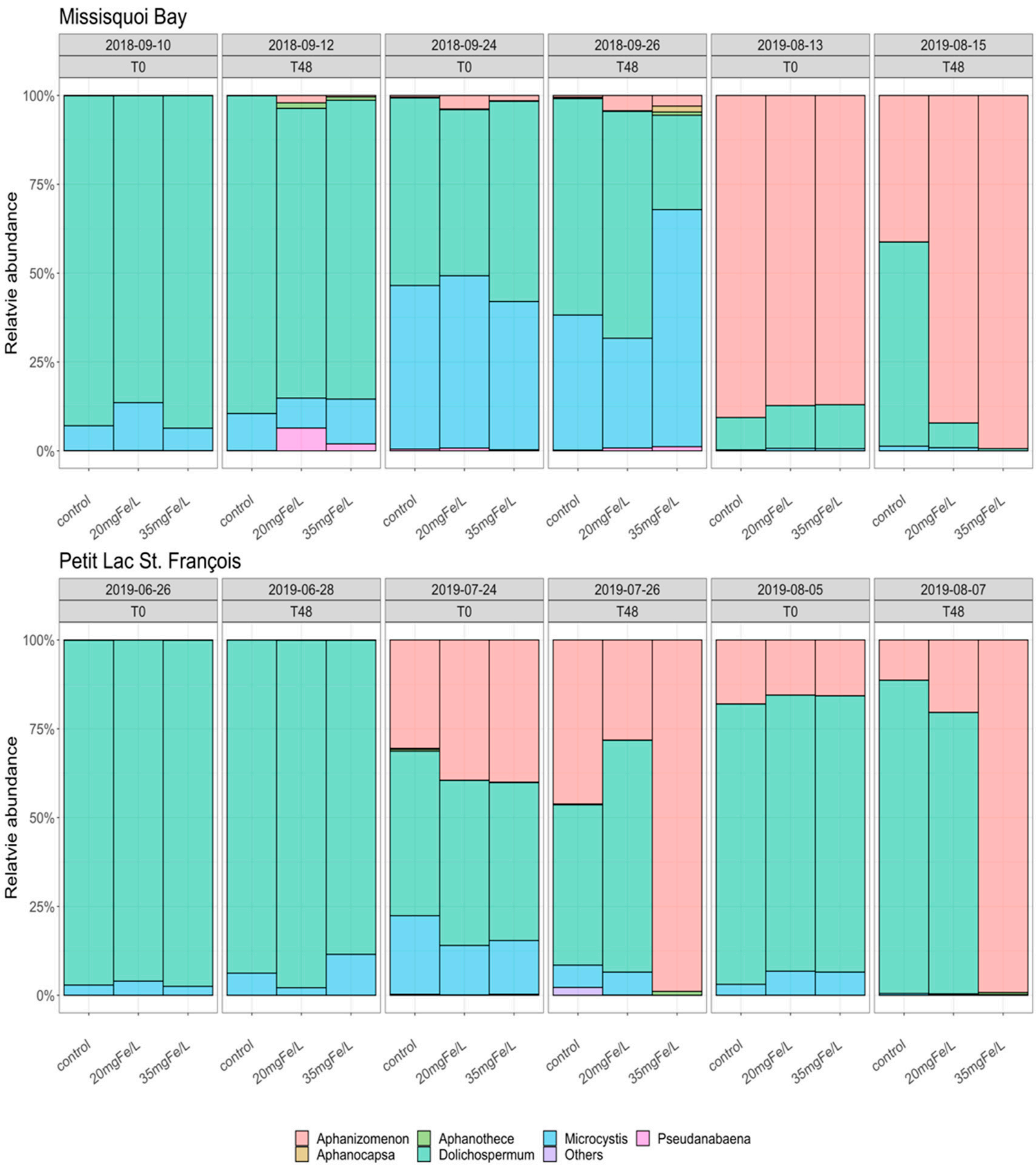

Figure S5. Relative abundance of cyanobacterial biovolume at genus level.

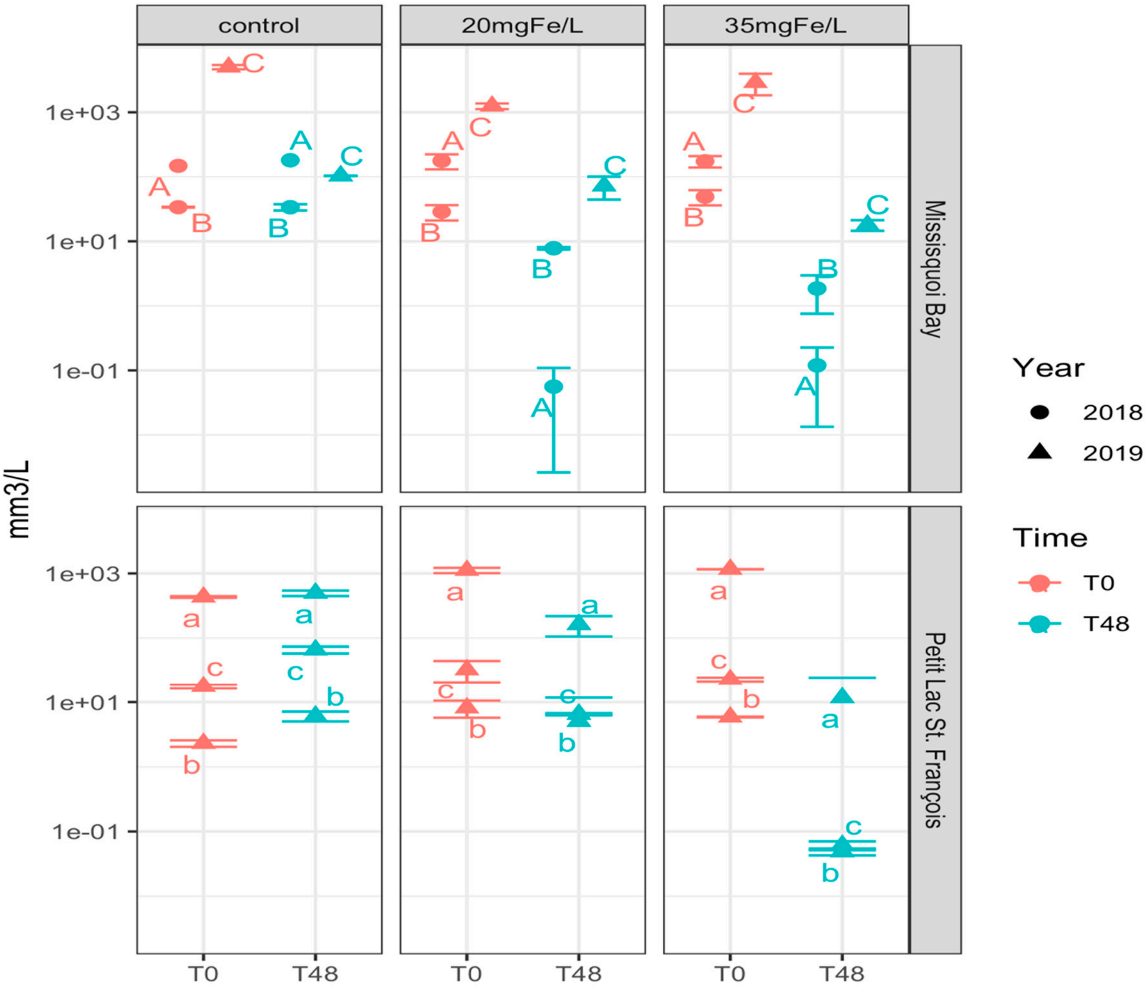

Figure S6. Total cyanobacterial biovolume (Mean ± standard deviation): A) September 10-12, 2018; B) September 24-26, 2018; C) August 13-15, 2019; a) June 26-28, 2019; b) July 24-26, 2019; c) August 05-07, 2019.

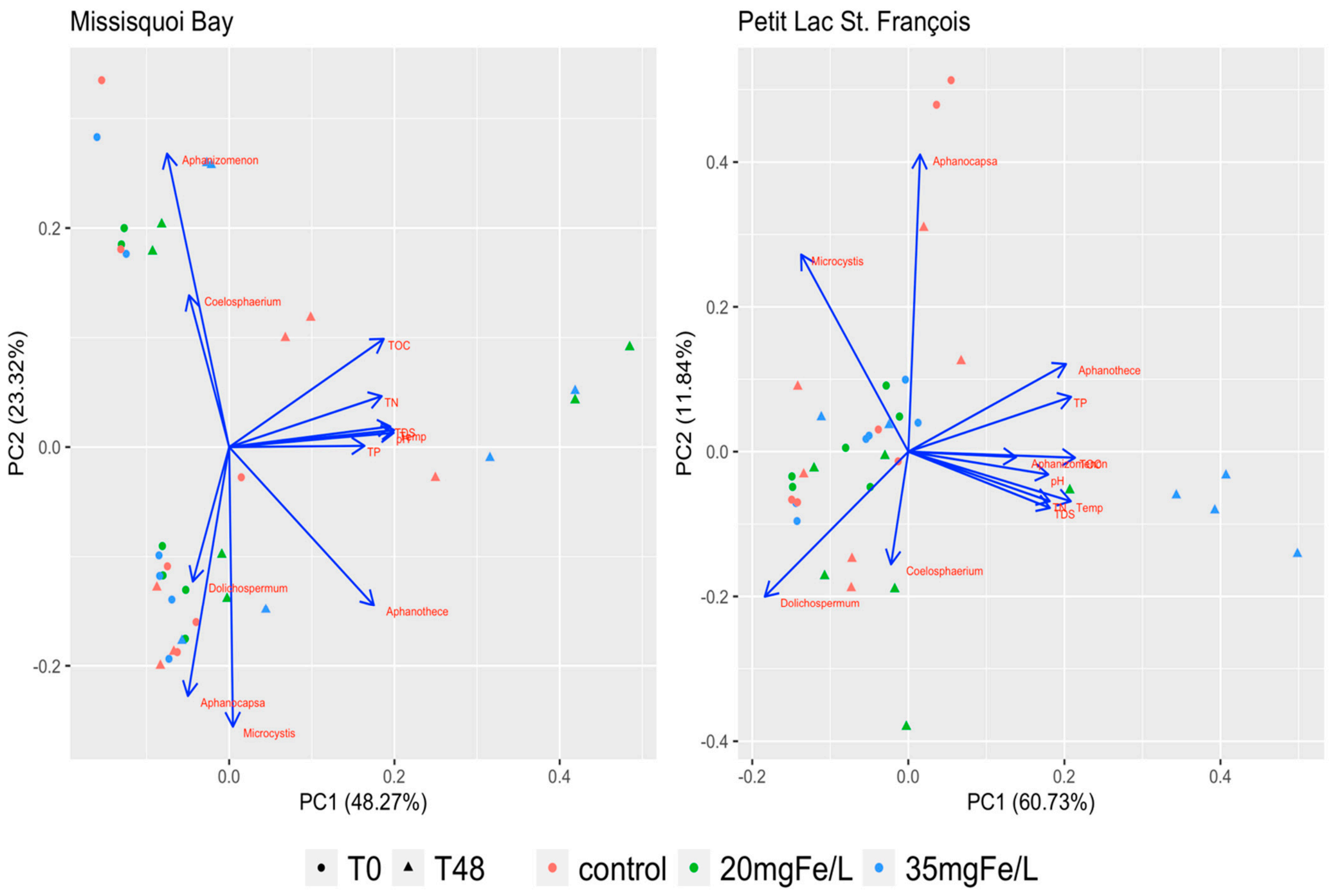

Figure S7. Principal component analysis (PCA) of cyanobacterial cell counts respect to environmental conditions in control, 20 mgFe/L and 35 mgFe/L mesocosms in Missisquoi Bay and Petit Lac St. François.

References

1. Merel, S.; Walker, D.; Chicana, R.; Snyder, S.; Baurès, E.; Thomas, O. State of knowledge and concerns on cyanobacterial blooms and cyanotoxins. *Environment International* **2013**, *59*, 303–327, https://doi.org/10.1016/j.envint.2013.06.013.
